# Childhood Interstitial Lung Disease in an Immunocompetent Patient Without Exposure

**DOI:** 10.7759/cureus.22266

**Published:** 2022-02-16

**Authors:** Saleh A Alharbi

**Affiliations:** 1 Pediatric Medicine, Umm Al-Qura University, Mecca, SAU

**Keywords:** inflammatory rheumatism, pulmonary function testing, chronic wet cough, fibrotic lung disease, pathogenesis ssc-ild

## Abstract

Childhood interstitial and diffuse lung disease (chILD) is a heterogeneous group of rare and chronic respiratory disorders with an estimated prevalence of 1.5 cases per million children aged 0-18 years. Various etiologies for chILD include but are not limited to systemic diseases, medications, exposure to tobacco, metabolic disorders, and organ diseases. Presented is the case of an immunocompetent young girl who presented with symptoms of recurrent cough and clubbing and was found to have interstitial lung disease.

## Introduction

Childhood interstitial and diffuse lung disease (chILD) is not a common entity of its own with a different grouping and a less well-known etiology. Further collaboration is needed to establish a clearer classification for chILD and advanced research is warranted to understand the complexity of the disease on the molecular and genetic levels [1].

## Case presentation

Presented is the case of a 14-year-old female of Southeast Asian origin, born and raised in Jeddah, Saudi Arabia. She originally sought medical advice with her non-consanguineous parents almost one decade ago at the age of 10 years. At the time of initial presentation, the complaint was recurrent cough and clubbing. Other than bronchial asthma, which was responsive to inhaled bronchodilator and low-dose inhaled corticosteroid treatment, she did not require any hospitalizations. She had received all her age-appropriate vaccines at the time of presentation, her perinatal history was unremarkable, and she had no family history of early death or similar illness. Physical examination at the time showed normal growth parameters and was not significant except for clubbing.

**Figure 1:**
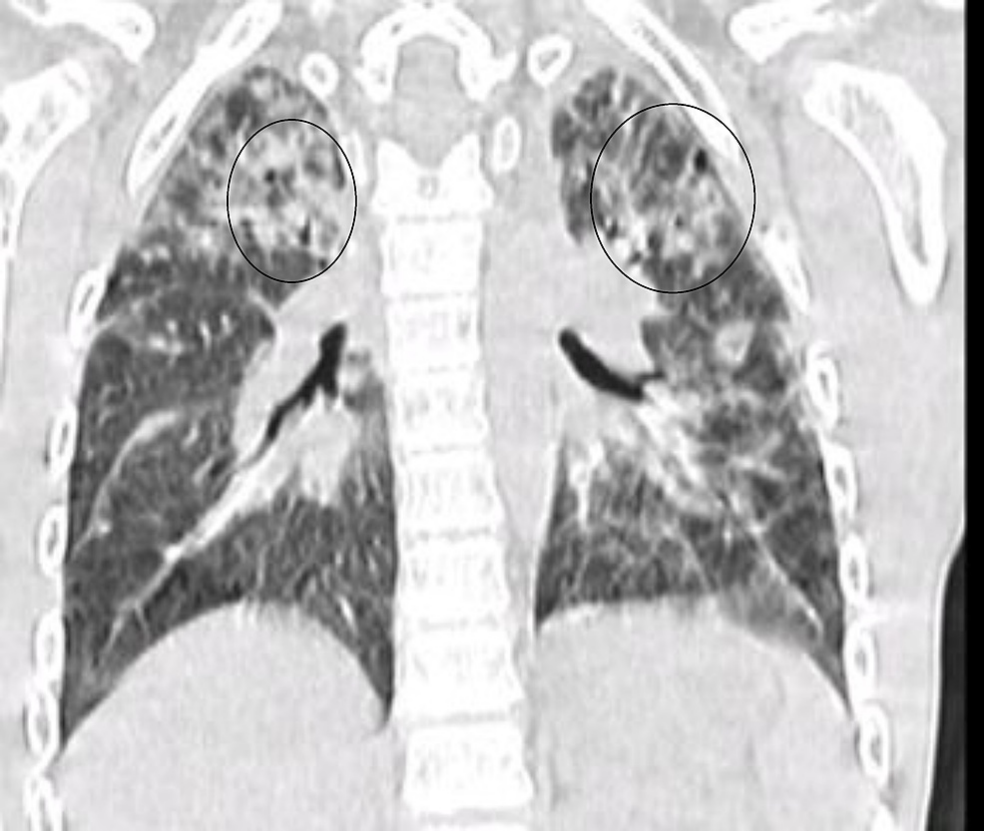
Coronal cut of high-resolution CT in 2011. High-resolution chest imaging showed a picture consistent with findings of interstitial lung disease.

Lab tests were negative for diseases such as cystic fibrosis, α1 antitrypsin deficiency, hepatitis B, hepatitis C, HIV, systemic lupus erythematosus, and rheumatoid arthritis, with normal antinuclear antibody (ANA), C3, and C4 levels, and her complete blood count, as well as serum immunoglobulins, were normal except for elevated immunoglobulin E (IgE), which was 1454.3 IU/ml. Pulmonary function tests were indicative of obstructive disease responsive to bronchodilators. High-resolution chest imaging showed a picture consistent with findings of interstitial lung disease (ILD) (Figure 1). Imaging of the paranasal sinuses was normal. Initial open lung biopsy was done and revealed findings of non-specific interstitial pneumonia with a desquamative interstitial pneumonic pattern.

**Figure 2:**
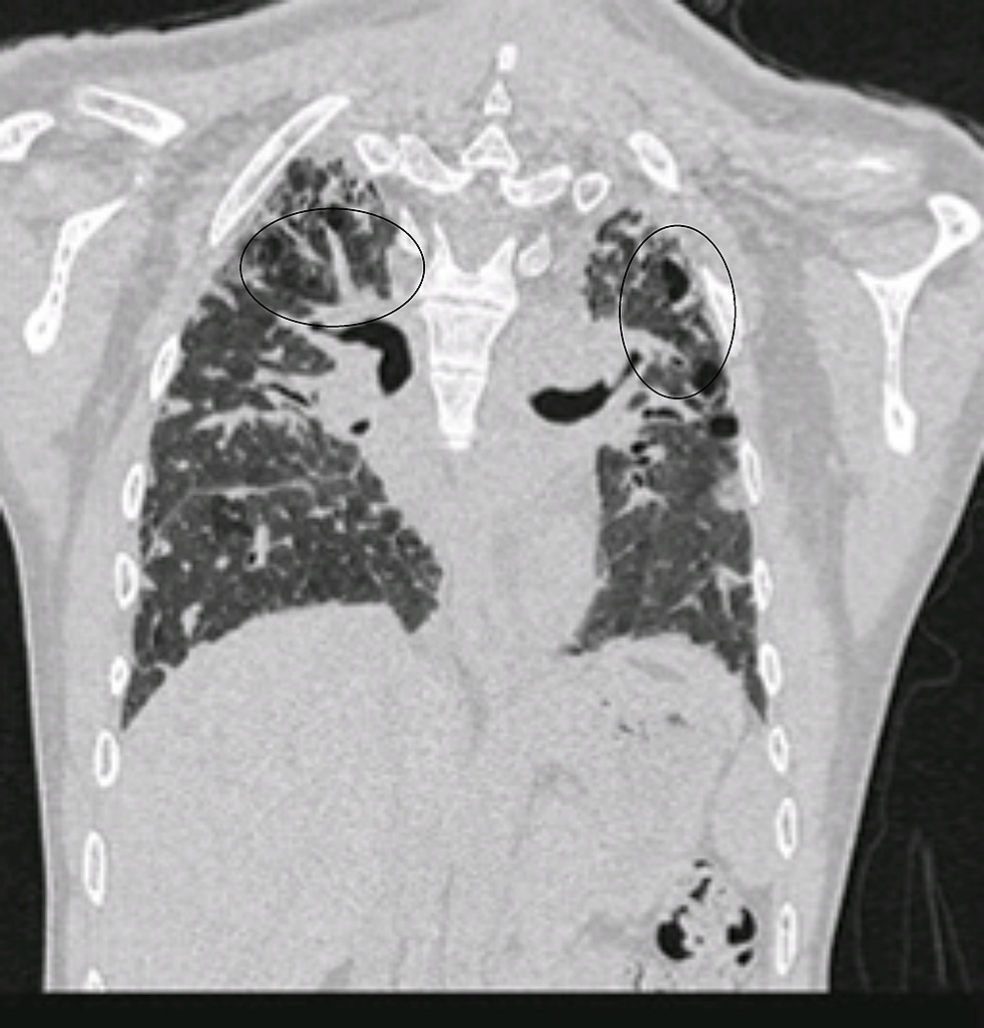
Coronal cut of high-resolution CT in 2018. Segmental areas of consolidation collapse were seen in both lung fields, especially in the posterior segment of the right upper lobe.

Over the past decade, she was symptomatically managed with oral and inhaled corticosteroids, anti-inflammatory drugs, mucolytic agents, intermittent home oxygen with a baseline pulse oxygen saturation of 89%, and antibiotics for episodes of pneumonia, with multiple admissions, two of which required intensive care admission. As her symptoms gradually worsened with increased dyspnea on exertion, her BMI decreased to 15 and her lung disease began to show signs of interval progression on high-resolution CT with an increase in the number and size of the bilateral pulmonary cystic air spaces with ground-glass opacities, septal thickening, bronchiectasis changes, and nodules. Segmental areas of consolidation collapse were seen in both lung fields especially in the posterior segment of the right upper lobe (Figure 2). Bronchoscopy was performed at the age of 17 and showed mainly inflammatory eosinophilic infiltrate. Serum IgE one year prior to bronchoscopy was still elevated at 824.2 IU/ml. Sputum culture was taken several times and was negative for bacterial infections. Testing for aspergillosis, mycobacteria, and atypical mycobacteria was also negative.

Her current management includes treatment for her lung disease with steroids, bronchodilators, mucolytics, antibiotics, and oxygen, being treated for microcytic hypochromic anemia, receiving preventive therapy for osteoporosis, and being given caloric support.

## Discussion

Asthma, cystic fibrosis, tracheomalacia, primary ciliary dyskinesia, persistent bacterial bronchitis, and even habitual cough are some of the causes of chronic cough, or cough lasting greater than four weeks, in the pediatric population [1]. Due to its rarity in children [2], another less well-known disease that may present in the pediatric age group with non-specific features of chronic cough and dyspnea on exertion is chILD [3]. The nomenclature in the literature regarding the disease differs, and terms such as diffuse lung disease or diffuse parenchymal lung disease have been used [3]. These terms are synonymous with a heterogeneous group of rare and chronic respiratory disorders with an estimated prevalence of 1.5 cases per million children aged 0-18 years [4].

Classification of chILD has proven to be challenging mainly due to its rarity in children and diagnosis may involve invasive procedures while the child or infant’s lung development must be taken into consideration on how it affects the disease process [2]. Several methods of classification have been proposed [5,6]. One classification divides ILD based on the age of presentation [5]. ILD that mainly develops in childhood includes diffuse developmental disorders of the alveoli, lung capillaries, and acini, stunted pulmonary growth, pulmonary interstitial glycogenosis, childhood neuroendocrine hyperplasia, and surfactant associated and unexplained respiratory distress of the mature newborn and the almost mature newborn [5]. ILD that can present at any age including those of pediatric age group, includes ILD associated with systemic disease, hypersensitivity or aspiration pneumonia in an immunocompetent individual, infection or bronchiolitis obliterans in an immunocompromised patient, structural vascular changes, reactive lymphoid lesions, and ILD of unclear respiratory distress syndrome in an older child [5]. A recent review of chILD published in 2018 reviewed 148 published articles between 2016 and 2017 and only two (1.4%) articles discussed ILD in immunocompetent patients that had some type of exposure [6]. None of the articles reviewed involved immunocompetent children without prior exposure or were previously well prior to presentation. chILD is very uncommon to occur spontaneously in an immunocompetent individual.

Various etiologies for chILD include but are not limited to systemic diseases such as systemic lupus erythematosus, rheumatoid arthritis, connective tissue disease and vasculitis, medications such as immunosuppressants and chemotherapeutics, exposure to tobacco, metabolic disorders such as lysosomal diseases, and organ diseases such as chronic hepatitis or celiac disease [7]. Eosinophilic lung disease may be associated with allergic bronchopulmonary aspergillosis and medication reactions, and ILD is present in 90% of children with eosinophilic granulomatosis associated with polyangiitis, although rare to occur in this age group [8]. Based on the histologic classification, there remains unclassified or idiopathic interstitial pneumonia in children [9]. Both desquamative interstitial pneumonitis and lymphocytic interstitial pneumonitis are commonly seen in adult smokers and those with immune defects or connective tissue diseases, respectively, and are rarely seen in children as well [9].

Children with ILD have a spectrum of clinical presentation ranging from a prolonged asymptomatic phase to recurrent cough, exertional dyspnea, and frequent chest infections [10]. Due to the wide clinical spectrum and rarity of chILD, diagnosis and management for this heterogeneous group of diseases depend on reports of other cases in the literature [11]. Diagnostic approaches other than high clinic suspicion may include pulmonary function tests, repeated chest X-rays, high-resolution chest CT, bronchoalveolar lavage, and lung biopsy [12]. Genetic testing may be useful with whole-genome sequencing especially with some gene mutilations that have been found to be linked with surfactant transport and mutations. These tests, however, need to be interpreted with caution and with the clinical context of the patient in mind given that not all mutations for chILD have been uncovered [13].

In chILD, a lung biopsy may assist with clinical management, especially for steroid dosing. On average, antibiotics, immune modulators, oxygen support, and the fraction of inspired oxygen (FiO2) did not differ significantly before and after biopsy, but the pathological evaluation gave diagnostic information that led to changes in therapeutic management in more than half of the population [13,14].

High morbidity and mortality are associated with chILD and treatments are varied [15]. The mainstay of treatment other than supportive therapy with oxygen, adequate nutrition, bronchodilators, and antibiotics for infections are corticosteroids for patients diagnosed with chILD [16]. Some have attempted the use of hydroxychloroquine and azathioprine, and the final treatment being offered for those with end-stage disease is lung transplantation [16].

## Conclusions

Childhood ILD is not a common entity of its own with a different grouping and a less well-known etiology. Further collaboration is needed to establish a clearer classification for chILD and advanced research is warranted to understand the complexity of the disease on the molecular and genetic level as well as to gather more knowledge regarding patients worldwide through international collaboration and databases. These efforts may one day enable future physicians and clinicians to have evidence-based practice guidelines to better manage patients with chILD.

## References

[1] Weinberger M, Fischer A (2014). Differential diagnosis of chronic cough in children. Allergy Asthma Proc.

[2] Nicholson AG, Bush A (2007). Classification of diffuse lung disease in infants: the reality of groups. Am J Respir Crit Care Med.

[3] Cunningham S, Jaffe A, Young LR (2019). Children's interstitial and diffuse lung disease. Lancet Child Adolesc Health.

[4] Saddi V, Beggs S, Bennetts B (2017). Childhood interstitial lung diseases in immunocompetent children in Australia and New Zealand: a decade's experience. Orphanet J Rare Dis.

[5] Länger F, Werlein C, Soudah B, Schwerk N, Jonigk D (2021). Interstitial lung disease in infancy and early childhood. (Article in German). Pathologe.

[6] Griese M (2018). Chronic interstitial lung disease in children. Eur Respir Rev.

[7] Clement A, Nathan N, Epaud R, Fauroux B, Corvol H (2010). Interstitial lung diseases in children. Orphanet J Rare Dis.

[8] Semple TR, Ashworth MT, Owens CM (2017). Interstitial lung disease in children made easier…well, almost. Radiographics.

[9] Fan LL, Deterding RR, Langston C (2004). Pediatric interstitial lung disease revisited. Pediatr Pulmonol.

[10] Nathan N, Berdah L, Delestrain C, Sileo C, Clement A (2020). Interstitial lung diseases in children. Presse Med.

[11] Deterding RR, DeBoer EM, Cidon MJ, Robinson TE, Warburton D, Deutsch GH, Young LR (2019). Approaching clinical trials in childhood interstitial lung disease and pediatric pulmonary fibrosis. Am J Respir Crit Care Med.

[12] Vijayasekaran D (2013). Interstitial lung disease in children. Indian Pediatr.

[13] Nogee LM (2006). Genetics of pediatric interstitial lung disease. Curr Opin Pediatr.

[14] Hafezi N, Heimberger MA, Lewellen KA, Maatman T, Montgomery GS, Markel TA (2020). Lung biopsy in children's interstitial and diffuse lung disease: does it alter management?. Pediatr Pulmonol.

[15] Hime NJ, Zurynski Y, Fitzgerald D (2015). Childhood interstitial lung disease: a systematic review. Pediatr Pulmonol.

[16] Fan LL, Langston C (1993). Chronic interstitial lung disease in children. Pediatr Pulmonol.

